# Retention Behavior of Anticancer Thiosemicarbazides in Biomimetic Chromatographic Systems and In Silico Calculations

**DOI:** 10.3390/molecules28207107

**Published:** 2023-10-16

**Authors:** Marek Studziński, Paweł Kozyra, Monika Pitucha, Bogusław Senczyna, Joanna Matysiak

**Affiliations:** 1 Department of Physical Chemistry, Institute of Chemical Sciences, Faculty of Chemistry, Maria Curie-Skłodowska University, 20-031 Lublin, Poland; marek.studzinski@umcs.pl; 2 Independent Radiopharmacy Unit, Medical University of Lublin, Chodzki 4a, 20-093 Lublin, Poland; pawelkozyra@umlub.pl (P.K.); monikapitucha@umlub.pl (M.P.); 3 Department of Chemistry, University of Life Sciences in Lublin, Akademicka 15, 20-950 Lublin, Poland; boguslaw.senczyna@up.lublin.pl

**Keywords:** HPLC chromatography, thiosemicarbazide, lipophilicity, plasma–protein binding

## Abstract

Chromatographic methods, apart from in silico ones, are commonly used rapid techniques for the evaluation of certain properties of biologically active compounds used for their prediction of pharmacokinetic processes. Thiosemicarbazides are compounds possessing anticancer, antimicrobial, and other valuable biological activities. The aim of the investigation was to estimate the lipophilicity of 1-aryl-4-(phenoxy)acetylthiosemicarbazides, to predict their oral adsorption and the assessment of their % plasma–protein binding (%PPB). RP-HPLC chromatographic techniques with five diversified HPLC systems, including columns with surface-bonded octadecylsilanes (C-18), phosphatidylcholine (immobilized artificial membrane, IAM), cholesterol (Chol), and α1-acid glycoprotein (AGP) and human serum albumin (HSA), were applied. The measured lipophilicity of all investigated compounds was within the range recommended for potential drug candidates. However, some derivatives are strongly bonded to HSA (%PPB ≈ 100%), which may limit some pharmacokinetic processes. HPLC determined lipophilicity descriptors were compared with those obtained by various computational approaches.

## 1. Introduction

Chromatographic methods, apart from in silico ones, are commonly used, quick techniques for the evaluation of some properties of biologically active compounds [1,2,3]. They are mainly used to assess lipophilicity and other biomimetic properties such as compound plasma–protein binding (PPB) [4]. The retention times of compounds in chromatography systems are directly proportional to the dynamic equilibrium partition constant between the mobile phase and the biological membrane or proteins as the stationary phases. This phenomenon is very similar to the absorption and distribution processes of drugs taking place in living organisms, which are non-equilibrated, dynamic processes. Therefore, various chromatographic methods are commonly used to model biological systems and determine quantities that describe the behavior of compounds in in vivo systems well [5,6,7,8]. They are commonly used for the determination of lipophilicity.

According to the IUPAC definition, “Lipophilicity represents the affinity of a molecule or a moiety for a lipophilic environment” [9]. In the context of drugs or drug candidates, it determines the ability of a molecule to penetrate biological membranes, i.e., the possibility of a molecule transporting in a passive way across the biological barrier such as a cellular membrane or the blood–brain barrier [10,11]. Taking into account the nature of membranes, the molecule should have a balanced lipophilic–hydrophilic character. On the one hand, the compound will dissolve in an aqueous environment, and on the other hand, this will enable the molecule to pass through biological membranes. Therefore, the lipophilicity parameter determines the adsorption and distribution processes of a compound in a living organism, but also affects metabolism and excretion. Considering the importance of this parameter, it is analyzed at a very early stage of research on potential drugs [2,3,4,12].

For conducting that type of investigation, column high-performance liquid chromatography (HPLC) seems to be the most widely used method. It enables the use of various commercially available stationary phases mimicking biological systems and delivers the most reproducible results. The HPLC was one of the techniques used to assess the lipophilicity of bioactive compounds. The evaluations of this parameter were performed in both isocratic [13] and gradient modes to optimize analysis time [14,15,16]. The comparative assessment of lipophilicity of structurally similar compounds is most frequently made using the log k_w_ parameter (logarithm of retention coefficient for water as mobile phase). In order to obtain values related to the classic extraction partition coefficient in the n-octanol-water system log Po/w, appropriate calibration curves are used [17]. The C-18 RP-HPLC method is officially recommended by the International Union of Pure and Applied Chemistry (IUPAC) and the Organization for Economic Co-operation and Development (OECD) as an alternative for the classic, time-consuming flask-shaking method. The octadecyl phase C-18 is commonly used as stationary phase, also but less frequently octyl C-8 [7], immobilized artificial membrane (IAM) [5,7] or cholesterol (Chol) phases [9,10] are applied. The mobile phase is a buffered (or not) water–organic modifier mixture. As organic modifiers, MeOH (C-18) or ACN (IAM) are most commonly used [11,14].

The main barrier to drug absorption and their distribution in the living organisms are fluid cell membranes. A better model of this biological system than the C-18 phase seems to be immobilized artificial membrane (IAM) columns. They were applied as a stationary phase for the first time by Pidgeon et al. [18,19]. The IAM surface was obtained by chemical bonding of phosphatidylcholine to a solid surface of silica gel. It mimics the density of phosphatidylcholine in the biological membrane bilayer. IAM columns are applied for the prediction of the oral absorption of drug candidates and their permeability through Caco-2 cells [20]. In this system in the retention mechanism, in addition to hydrophobic interactions, ionic interactions take place; this is particularly significant in the case of ionizable compounds [11,18,21].

The cholesterol stationary phase is another one used for the lipophilicity assessment [13,14,22]. In this case, the retention mechanism, similar to that in the case of the C-18 column, is based mostly on hydrophobic interactions and partition mechanism; thus, the same composition of mobile phases can be applied for lipophilicity determination. However, especially in cases of π-electron-containing molecules, some differences can be observed in comparison to the octadecyl phase. The advantage of this phase in comparison to C-18 is that, during the use of mobile phases containing high concentrations of water, no “phase collapse” effect has been observed due to the strong repulsion of non-polar surface groups by water [23].

In recent years, columns with immobilized proteins have been introduced, allowing for the evaluation of drug (compound)–plasma–protein binding strength, mainly with human serum albumin (HSA) and α1-acid glycoprotein (AGP) [4,24]. HSA is the main plasma–protein responsible for binding drugs, and in many cases, it determines the drug–plasma–protein binding (PPB) ratio. This method correlates well with the conventional ultrafiltration one [25]. PPB has a significant impact on the concentration of the free (active) form of the drug in the plasma. This phenomenon has significant importance for the effectiveness of pharmacotherapy, and influences pharmacokinetics (i.e., distribution, clearance, and elimination half-life) and pharmacodynamics, i.e., efficacy and toxicity including drug–drug interactions [3,25,26,27].

The aim of the presented investigation was the lipophilicity estimation of 1-aryl-4-(phenoxy)acetylthiosemicarbazides to predict their oral absorption, and the assessment of % plasma–protein binding (% PPB) of these compounds. Thiosemicarbazides are described as anticancer agents [28,29,30,31]. Some of them target topoisomerase II alpha and indoleamine-2,3-dioxygenase 1 (IDO 1) [29], others induce apoptosis in cancer cells via JNK signaling in human breast cancer cells [30]. For the evaluation of considered compounds, RP-HPLC chromatography with five diversified HPLC systems including columns with octadecylsilanes (C-18), phosphatidylcholine (IAM), and cholesterol, as well as immobilized human serum albumin (HSA) alpha(1)-glycoprotein (AGP), were used. The parameters obtained by the chromatographic method were compared with those obtained by calculation methods using various algorithms. Correlation analysis and principal component analysis (PCA) were performed to evaluate obtained results.

## 2. Results and Discussion

### 2.1. Set of Analyzed Compounds

**Figure 1:**
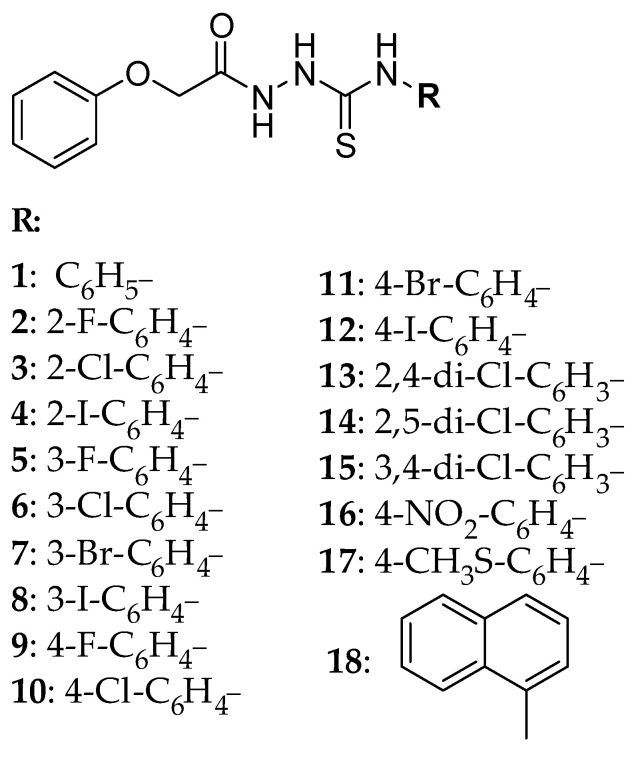
Structure of the tested compounds (**1**–**18**).

The retention behavior of 1-aryl-4-(phenoxy)acetylthiosemicarbazides presented in Figure 1 was investigated by HPLC under isocratic conditions. Compounds **1**–**18** were obtained in the reaction of phenoxyacetic acid hydrazide with the appropriate isothiocyanate at the boiling point of MeOH. The anticancer potential of the compounds against prostate and melanoma human cancer cells has been proved [31].

### 2.2. HPLC Lipophilicity and % PPB

For the evaluation of the compounds the following stationary phases were used: C-18; biomimetic, IAM; cholesterol (Chol); immobilized proteins HSA and AGP. For the lipophilicity determination, MeOH (C-18, Chol) or ACN (IAM) were applied as the organic modifiers. The measurements were performed at pH 7.4 of the mobile phase. The regular changes in the retention of all solutes used as organic modifier content in the mobile phase on three studied stationary phases were found. It is expressed by the Soczewiński–Wachtmeister Equation (1) [32]:

log k = log k_w_ + S (% organic modifier) (1)

where log k_w_—the intercept; S—the slope of regression curve. The log k_w_ values are usually obtained by linear extrapolation, since the vast majority of compounds do not migrate in pure water as the mobile phase [33,34]. Log k_w_ as well as S parameters are commonly applied as lipophilicity descriptors [22,34]. The obtained results are included in Table 1.

**Figure 2:**
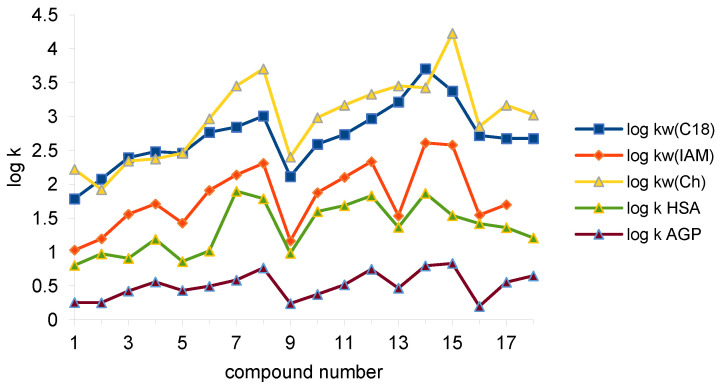
Retention of the investigated compounds on C-18, IAM, Chol, and IAM (log k_w_) and on HSA and AGP stationary phases (log k, 15% propan-2-ol).

Measurements on HSA and AGP immobilized protein columns were carried out with 0.15% isopropanol content in the aqueous mobile phase. The results are presented in Table 2. The comparison of the retentions of the investigated compounds are presented in Figure 2 and Table 3.

The series of mean retention log k_w_ from the highest to the lowest presents as follows: Chol > C-18 > IAM and log k as: HSA > AGP. The greatest diversity of log k_w_ was obtained for the Chol column and the lowest was obtained for the IAM one; this is similarly the case for the mean values. The order of (-S) values is as follows: IAM > Chol > C-18. Standard deviation for C-18 and IAM columns are similar but the octadecyl phase covers the greatest range. Higher log k_w_ values on C-18 than on IAM were observed for many various groups of compounds [22,33,36].

The lowest log k_w_ values (except Chol) are observed for unsubstituted compound **1**. The retention of *orto*, substituted in phenyl ring analogs, is usually lower compared to other isomers. *Meta*- and *para*-substituted derivatives have comparable retention. In the case of Chol, the column retention of the *meta*-substituted derivatives is slightly higher than the *para*-substituted derivatives. The retention of fluorinated derivatives substituted in the same position increases with the atomic weight increase in the substituent. In the case of dichloro-analogs (**13**, **14**, **15**), the highest retention of some compounds can be observed. The lipophilicity of compounds with complex substituents is higher than unsubstituted parent compound (**1**).

The second group of stationary phases used in the experiment belongs to the group of immobilized protein columns HSA and AGP; this allows us to assess the ratio of compounds binding to the plasma–proteins. HSA and AGP are the main proteins present in the plasma of human blood. Free-form drugs in plasma undergo pharmacokinetic processes and their concentrations are responsible for the observed pharmacological effect. The molecules of drugs bonded to plasma–protein are not available for distribution processes, hepatic metabolism, or elimination [37]. Therefore, this property is determined at the initial stage of research into potential drugs.

The standard calibration curve was used to predict log K values (K—binding equilibrium constant) of investigated compounds to HSA and AGP. The results are presented in Table 2. Next, the log K values were converted into a percentage of plasma–protein binding (%PPB) of the compounds (Table 2). The results show that the compounds bind strongly to plasma–proteins, especially to HSA. %PPB to HSA is >93.5% and it is the lowest for the unsubstituted compound **1**, and the highest for 3-Br, 3-I, 4-Br, 4-I, and dichloro derivatives. The binding degree of compounds to AGP is significantly lower and ranges from about 70% to 91%. It is the lowest for compound **16** with -NO_2_ substituent, unsubstituted compound **1,** and fluorine derivatives **2** and **9**. In a group of fluorinated derivatives in the same position of the phenyl ring, the %PPB to AGP increases with the increase in the atomic weight of the halogen atom and the lipophilicity of the molecule.

### 2.3. Correlation Analysis

The estimated log k_w_ values on the three stationary phases are significantly different but they are correlated (Table 2, Figure 2). The following equations describing the relationship between the log k_w_ values on different stationary phases were obtained:

log k_w_ IAM = −0.7373 (±0.2130) + 0.9604 (±0.0778) log k_w_ C-18 (2)

$$n=17,\;R=0.9541,\;R^{2}=0.9104,\;R_{adj}^{2}\;=\;0.9045,\;F(1,15)\;=\;152.44\;p<0.00000,\;s\;=\;0.14967$$

Compound **16** is an outlier. The obtained log k_w_ IAM value is too low compared to C-18.

log k_w_ Chol = 0.0206 (±0.4552) + 1.0959 (±0.1662) log k_w_ C-18 (3)

$$n=17,\;R=0.8622,\;R^{2}=0.74345,\;R_{adj}^{2}\;=\;0.7263,\;F(1,15)\;=\;43.468\;p<0.00001,\;s\;=\;0.31982$$

Compound **16,** with the -NO_2_ substituent, is an outlier. It possesses lower log k_w_ Chol value compared to C-18.

log k_w_ Chol = 0.9137 (±0.2624) + 1.1197 (±0.1350) log k_w_ IAM (4)

$$n=18,\;R=0.8963,\;R^{2}=0.8033,\;R_{adj}^{2}\;=\;0.7910,\;F(1,16)\;=\;65.358\;p<0.00000,\;s\;=\;0.2715$$

Taking into account the slope and the intercept of the obtained equations (Equations (2)–(4)), it can be concluded that the most similar in terms of magnitude are the values of the log k_w_ obtained on the cholesterol and octadecyl phases (Equation (3)). The most diverse ones are those obtained on the IAM and cholesterol phases. However, the best correlation for log k_w_ IAM and log k_w_ C-18 parameters was found. In other cases, the correlations are slightly weaker. According to Ong and Pidgeon, the partitioning process is the principal retention mechanism in the IAM retention and includes both hydrophobic and polar interactions with the solvated layer(s) of the stationary phases and ionizable groups of immobilized phospholipids [19]. Therefore, the obtained log k_w_ IAM values are different, in this case lower, compared to the log k_w_ C-18, where the hydrophobic interactions determine the retention [11].

Lipophilic log k_w_ descriptors obtained by chromatographic methods are often compared with those obtained by calculation methods (log P, log D) [6,7]. They are even faster in estimation and allow us to predict this parameter even for virtual compounds. This is of particular importance in the design and synthesis of bioactive compounds. The log P (log D) coefficients obtained with several calculation methods are summarized in Table 4.

**Figure 3:**
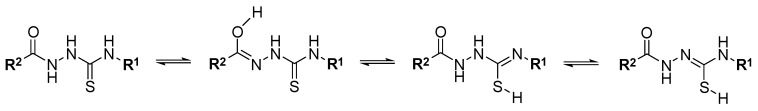
Some tautomeric forms of thiosemicarbazides.

Table 5 presents the correlation matrix between the log k_w_ parameters and log P (log D) calculated using different approaches. The following best correlating pairs between chromatographic and in silico methods were found: the log k_w_ C-18 descriptors–M log P; the log k_w_ IAM–log P; log k_w_ Chol–log P (M-K) model. There was no increase in the correlation for the log D parameter compared to log P, although the determination of the chromatographic lipophilicity parameters of log k_w_ were performed at pH 7.4. The low correlation of log P (log D) parameters with log k_w_, even after the elimination of outlier compounds, indicates that in the case of the considered thiosemicarbazides, fast in silico methods do not provide satisfactory results, and experimental measurements (HPLC) are advantageous. This may be related to the existence of equilibrium tautomeric forms for the considered compounds, which is the result of migration of labile protons from –NH– groups into carbonyl (=C=O) or thiocarbonyl (=C=S) groups (Figure 3) [41,42].

This phenomenon is not taken into account in the case of calculation methods, but it was revealed in chromatographic analysis as well as biological systems. The participation of individual tautomeric forms, on the one hand, will be determined by the structure of the compound and electronic properties of molecules, on the other hand, by environmental conditions, such as the type of solvent, the pH, the temperature, etc. [41,42].

The lipophilicity of considered thiosemicarbazides is within the range recommended for potential drug candidates, for which favorable pharmacokinetic processes such as absorption or distribution after oral administration are predicted. The predicted Clog P values are less than 5, according to the Lipinski Rule of Five [43], and the Mlog P values ranging from −2.0 to 4 take into account the Oprea recommendations [44]. The obtained log P values are far from the limit, thus, even taking into account the participation of isomeric forms—for which log P will have different values compared to the basic form—it may be assumed that they will still fall within the recommended range.

**Figure 4:**
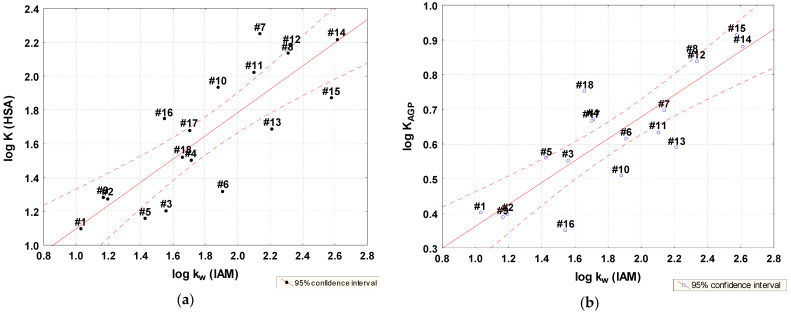
Relationships between lipophilicity parameter log k_w_ IAM and log K for HAS (**a**) and AGP (**b**).

Significant statistical correlations were found between the log K parameter and lipophilicity and the best models were obtained for lipophilicity expressed by log k_w_ IAM (Figure 4, Equations (5) and (6)). 

This confirms the thesis that neutral molecules are strongly bound to HSA and the fact, that for some groups of compounds the binding to HSA is dominated by their lipophilicity. In the case of the studied thiosemicarbazides, lipophilicity also determines the affinity to AGP [26,45].

log K_HSA_ = 0.40976 (±0.2181) + 0.68721 (±0.1151) log k_w_ IAM (5)

$$n=18,\;R=0.8307,\;R^{2}=0.6901,\;R_{adj}^{2}\;=\;0.6708,\;F(1,16)\;=\;41.07\;p<0.00002,\;s\;=\;0.2256$$

log K_AGP_ = 0.04690 (±0.0932) + 0.31540 (±0.0492) log k_w_ IAM (6)

$$n=18,\;R=0.8483,\;R^{2}=0.7197,\;R_{adj}^{2}\;=\;0.6708,\;F(1,16)\;=\;35.64\;p<0.00009,\;s\;=\;0.3198$$

It is generally accepted that %PPB to HSA correlates well with the conventional ultrafiltration method and describes the overall drug–plasma binding process well, although both methods have their advantages and limitations. Other individuals responsible for protein binding should also be considered as very important in some cases. However, in terms of the %PPB to AGP measurements using immobilized columns and their interpretation, opinion is divided [4,25]. 

**Figure 5:**
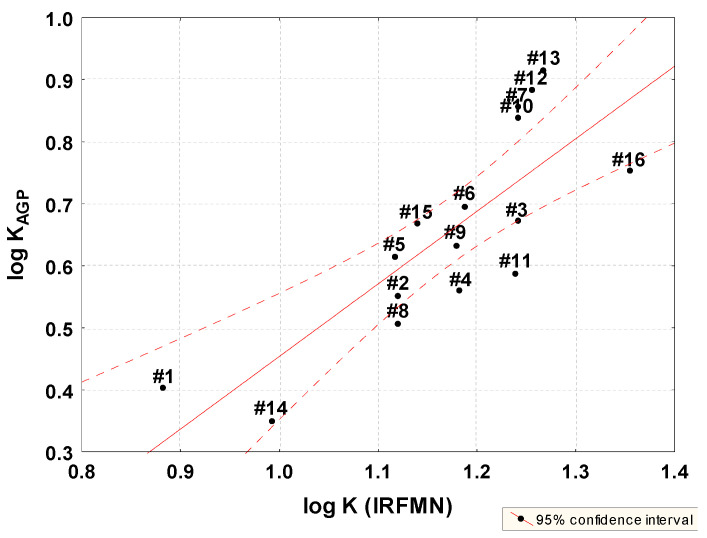
Relationship between PPB log K (IRFMN) predicted and estimated by chromatography log K AGP.

Plasma–protein binding values with log K RFMN were also calculated using Vega QSAR software v. 1.15 beta 47 (IRFMN prediction, a-dimensional) (Table 2) [35]. A correlation was found between the predicted values and the chromatographic log K_AGP_ (Figure 5, Equation (7)). In the case of the log K_HSA_ parameter, this relationship is weaker (r = 0.43).

log K_AGP_ = −0.7158 (±0.2724) + 1.1697 (±0.2313) log K (IRFMN) (7)

$$n=16,\;R=0.8038,\;R^{2}=0.6462,\;R_{adj}^{2}\;=\;0.6209,\;F(1,14)\;=\;25.57\;p<0.00018,\;s\;=\;0.1017$$

Compounds **2** and **9** are outliners. Log K (IRFMN) is also highly correlated with in silico lipophilicity parameters (r in the range from 0.83 to 0.94), in particular with log P (M-K) model (Table 5). 

### 2.4. PCA Analysis

**Figure 6:**
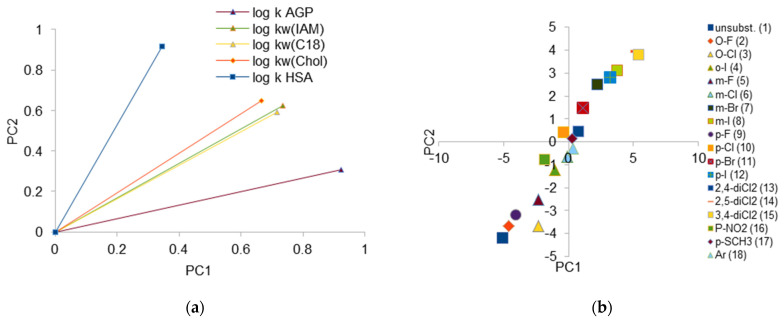
(**a**) PCA loading plot for logarithm of retention coefficient (log k_w_) for investigated chromatographic system. (**b**) PCA score plot of retention data obtained for investigated compounds.

A principal component analysis (PCA) was also carried out to compare all chromatographic systems. PCA chromatographic data for investigated compounds can be practically reduced to one parameter (Eigen values for two largest components are equal 4.196 and 0.360, respectively). But in order to conduct a detailed investigation of the obtained data, a two-dimensional analysis was performed. The method used for rotation is Varimax; the loading plot for log k is depicted in Figure 6a and score plot is depicted in Figure 6b.

Figure 6 confirms strong correlations for IAM, C-18, and Chol columns. This proves that the retention mechanism in those systems is generally similar; however, some differences can be observed. The greatest similarity for the IAM and C-18 phases is found, unlike in the case of the correlation analysis, where the greatest similarity for the log k_w_ parameters obtained on the IAM and Chol phases was stated. The biggest differences can be observed for AGP and HSA columns; this proves that, in both cases, the retention of solutes is additionally influenced, most probably by steric phenomena on surface of the protein-modified stationary phase. The specific affinitive retention mechanism is also involved. The placement of loading vectors also suggests that retention of investigated compounds on HSA differs significantly from the one on AGP. 

A score plot presenting the behavior of the compounds in all the investigated chromatographic systems is presented in Figure 6b. The first thing which can be observed is the fact that unsubstituted compound **1** has different chromatographic properties than the others. In spite of these differences, compounds containing fluorine atom regardless of its location (*meta*-, *ortho*-, *para*-) lie on the graph closely to each other and to unsubstituted compound. In the case of I- and Cl- derivatives, *orto*- ones differ significantly from the others. What can be explained as the effect of lower electron affinity (larger volume and higher hydrophobicity) of those atoms. Similarly, in the cases of dichloro derivatives, the difference between the chromatographic behavior of 2,4-disubstituted isomers and other isomers can be observed. Compound **18** with naphthyl ring, *m*-Cl, *p*-Cl, and *p*-SCH_3_ derivatives presents the most similar (and closest to average) chromatographic properties, regardless of the system used.

## 3. Materials and Methods

### 3.1. HPLC Measurements

HPLC measurements were performed using a liquid chromatograph Knauer (Knauer, Berlin, Germany) with a dual pump, a 20 µL simple injection valve, and a UV–visible detector. The compounds were detected under UV light at 280 nm at room temperature. The retention time of an unretained solute (t_0_) was determined by the injection of a small amount of citric acid dissolved in water.

### 3.2. C-18 Chromatography

In the RP-18 HPLC chromatography process, the Eurosil Bioselect C-18 (5 μm, 300 × 4.6 mm) column was used as the stationary phase. The mobile phase consisted of different volume mixtures of MeOH as the organic modifier and 20 mM acetate buffer as the aqueous phase, to obtain pH = 7.4. The MeOH concentration ranged from 0.4 to 0.9 (*v*/*v*), at 0.1 intervals (Table S1 in the Supplementary Materials). Details are provided in Kozyra et al. [31].

### 3.3. IAM Chromatography

A Rexchrom IAM.PC.DD2 (12 μm, 100 × 4.6 mm, 300 Å) (Regis Technologies, Morton Grove, IL, USA) column was used as the stationary phase. The compounds were dissolved at a concentration of 0.5 mg × mL^−1^ in MeOH. The mobile phases consisted of different volume fractions of ACN and 20 mM phosphate buffer; the aqueous phase maintained a pH = 7.4 (0.02 M KH_2_PO_4_, Na_2_HPO_4_ and 0.15 M KCl). The acetonitrile concentration ranged from 0.05 to 0.4 (*v*/*v*), depending on the structure of compound, at 0.05 intervals (Table S2 in the Supplementary Materials). The flow rate was 1 mL × min^−1^. 

### 3.4. Cholesterol Chromatography

Cogent 4 UDC Cholesterol (150 × 2.1 mm, 4 µm) MicroSolv Technology Corporation (Leland, NC, USA) column was used. The mobile phase consisted of different volume mixtures of MeOH as the organic modifier and 20 mM acetate buffer as the aqueous phase, to obtain pH = 7.4. The concentrations of the organic modifier were in the range from 0.3 to 0.8 (*v*/*v*) with step 0.05 or 0.1 (Table S3 in the Supplementary Materials). The flow rate was 0.35 mL × min^−1^.

### 3.5. HSA and AGP Chromatography

A human serum albumin (HSA) immobilized on the 5 µm silica gel column 100 × 3 mm (Chiralpac) and an α_1_-acid glycoprotein (AGP) immobilized on the 5 µm silica gel column 100 × 4 mm (Chiralpac) were used. The compounds were dissolved at 0.5 mg/mL concentration in 50% propan-2-ol and ammonium acetate solution (pH = 7.4) mixtures for HSA measurements. The mobile phase was composed of 50 mM ammonium acetate solution (pH = 7.4) and propan-2-ol at 85/15 (*v*/*v*) for AGP, respectively. The flow of mobile phases was 0.5 mL×min^−1^. Log k values for the selected mobile phase were determined for all studied compounds and standard substances. The % protein plasma binding (% PPB) values were calculated from the calibration curve according to Valko et al. [4,45].

Calibration of the protein columns: The column performance check and the calibration check were performed before measurements. The racemic mixture of warfarin was used for their performance evaluation. The following calibration set of drugs was applied: bromazepam, carbamazepine, diclofenac, nicardipine, nizatidine, piroxicam for HSA and bromazepam, chlorpromazine, imipramine, nicardipine, nizatidine, propranolol, and warfarin for AGP. The analytical standards were purchased from Sigma Aldrich Steinhen, Steinheim am Albuch, Germany). The drugs were dissolved at 0.5 mg/mL concentration in 50% propan-2-ol and ammonium acetate solution mixtures (pH = 7.4). The obtained log k values from HPLC were plotted against the log K values (K—binding equilibrium constant, log K—linearized PPB) based on the literature data for plasma–protein binding (%, PPB). The following relationships were obtained:

log K_HSA_ = 0.2513 (±0.0984) + 1.0525 (±0.1171) log k HSA (8)

$$n=6,\;R=0.9761,\;R^{2}\;=\;0.9528,\;R_{adj}^{2}\;=\;0.9410,\;s\;=\;0.1948$$

log K_AGP_ = 0.1733 (±0.0574) + 0.8902 (±0.0787) log k AGP (9)

$$n=7,\;R=0.9810,\;R^{2}\;=\;0.9623,\;R_{adj}^{2}\;=\;0.9548,\;s\;=\;0.1113$$

Log K values were converted into %PPB using the following equation [15]:

(10) $$\%\mathrm{PPB}=\frac{101\times 10^{logK}}{1+10^{logK}}$$

### 3.6. Computational Methods

Clog P and log P values were calculated using the ChemDraw Ultra version 10.0 according to the fragmentation method introduced by Ghose and Crippen [38,46]. The estimations of S + log D and S + log P were made by the MedChem Designer (TM) version 3.0.0.30 [40]. The Moriguchi Mlog P, ALog P, log K, and %PPB were calculated by Percepta [39]. Log P (M-K) (Meylan-Kowwin) was calculated by Vega QSAR [35]. Statistica version 7.1 was used for the regression and correlation analysis [47] and JASP version 0.17.3 for PCA [48]. 

## 4. Conclusions

Chromatographic investigation involving various stationary and mobile phases modeling biological systems and calculation algorithms proved that the thiosemicarbazides used here are in the range of lipophilicity which is advised to be suitable for compounds to be considered as potential drug candidates. This highlights the high probability of a favorable absorption process, which is a critical property of orally administered drugs. Taking into account the low correlations between the log k_w_ and log P values of the investigated group of compounds, the application of chromatographic methods for detailed lipophilicity analysis of thiosemicarbazides is better justified compared to in silico ones. In the chromatographic systems, the contribution of the probable tautomeric forms may influence the retention processes. It is also probable that tautomers may be present in real biological processes. 

The large number of investigated compounds have a high level of human plasma–protein binding ratio, which may limit processes of drug distribution and slow down the metabolism and excretion of the investigated thiosemicarbazides. It is important to note that a high binding ratio can be observed for derivatives with the highest lipophilicity. In silico protein-binding-prediction methods were well correlated with obtained chromatographic parameters on the AGP column. However, weak correlations were observed for the albumin-bonded column; this is unsatisfactory due to the fact that most of the drugs in plasma bind to HSA.

## Figures and Tables

**Table 1:** (-S) and log k_w_ parameters of the Soczewiński–Wachtmeister equation (Equation (1)) obtained by HPLC chromatography on C-18, IAM, and Chol stationary phases.

No.	-S C-18 ^1^	log k_w_ C-18 ^1^	r^2^	-S IAM	log k_w_ IAM	r^2^	n	-S Chol	log k_w_ Chol	r^2^	n
**1.**	3.7611	1.7866	0.9937	4.4559	1.0288	0.9390	8	4.0307	2.2209	0.9947	7
**2.**	4.3603	2.0774	0.9955	4.8953	1.1961	0.9931	7	3.7822	1.9241	0.9952	6
**3.**	4.5312	2.3929	0.9973	5.2422	1.5588	0.9818	6	4.1573	2.3437	0.9917	7
**4.**	4.4182	2.4824	0.9986	5.3942	1.7099	0.9810	6	4.1215	2.3754	0.9959	7
**5.**	4.7921	2.4592	0.9968	5.5312	1.4283	0.9955	7	4.3726	2.4598	0.9918	6
**6.**	4.8532	2.7672	0.9978	6.3395	1.9101	0.9929	6	4.8849	2.9670	0.9916	6
**7.**	4.8454	2.8451	0.9973	6.8947	2.1389	0.9847	5	5.4314	3.4508	0.9878	6
**8.**	4.9651	3.0072	0.9972	6.9157	2.3083	0.9871	8	5.6623	3.7019	0.9747	6
**9.**	4.2984	2.1145	0.9869	5.1370	1.1652	0.9629	7	4.2398	2.4029	0.994	7
**10.**	4.5132	2.5914	0.9977	6.0976	1.8779	0.9920	6	4.8387	2.9842	0.9936	8
**11.**	4.6183	2.734	0.9964	6.5856	2.1020	0.9781	5	5.0251	3.1674	0.9925	6
**12.**	4.8352	2.9681	0.9957	6.8364	2.3341	0.9871	6	5.0829	3.3300	0.9908	6
**13.**	5.1803	3.2152	0.9973	6.2034	1.5351	0.9811	6	5.4505	3.4517	0.9802	
**14.**	5.6376	3.7050	0.9976	7.6211	2.6121	0.9853	6	5.1337	3.4243	0.9624	5
**15.**	5.3305	3.3757	0.9991	7.5617	2.5780	0.9826	6	6.2128	4.2283	0.9626	6
**16.**	5.1865	2.7200	0.9905	6.0875	1.5455	0.9942	7	5.2122	2.8538	0.9748	6
**17.**	4.6859	2.6753	0.9990	5.7908	1.6998	0.994	6	5.0386	3.1664	0.9782	6
**18.**	4.6919	2.6769	0.9992	5.6500	1.6606	0.9903	6	5.0400	3.0227	0.9965	6

^1^—The values were taken from Kozyra et al. [31].

**Table 2:** Affinity of the compounds to HSA and AGP (log k and log K), %PPB (% plasma–protein binding) determined by HPLC and predicted affinity to proteins—log K (IRFMN).

No.	log k HSA	log K HSA	%PPB HSA	log k AGP	log K AGP	%PPB AGP	log K IRFMN ^1^
**1.**	0.8050	1.0986	93.5	0.2567	0.4018	72.3	0.8835
**2.**	0.9750	1.2775	95.9	0.2531	0.3986	72.2	1.1352
**3.**	0.9070	1.2059	95.1	0.4257	0.5523	78.9	1.1203
**4.**	1.1899	1.5037	97.9	0.5588	0.6707	83.2	1.2404
**5.**	0.8615	1.1581	94.4	0.4328	0.5586	79.1	1.1834
**6.**	1.0170	1.3217	96.4	0.4964	0.6152	81.3	1.1166
**7.**	1.8991	2.2501	100	0.5881	0.6968	84.1	1.1875
**8.**	1.7890	2.1342	100	0.7664	0.8556	88.6	1.2404
**9.**	0.9823	1.2852	96.0	0.2424	0.3891	71.7	1.1352
**10.**	1.5981	1.9333	99.8	0.3754	0.5075	77.0	1.1203
**11.**	1.685	2.0248	100	0.518	0.6344	82.0	1.178
**12.**	1.8304	2.1778	100	0.7465	0.8378	88.2	1.2404
**13.**	1.3649	1.6879	99.0	0.4665	0.5886	80.3	1.2395
**14.**	1.8689	2.2183	100	0.7971	0.8829	89.3	1.2545
**15.**	1.5391	1.8712	99.7	0.8332	0.9150	90.1	1.2677
**16.**	1.4198	1.7456	99.2	0.1994	0.3508	69.8	0.9916
**17.**	1.3603	1.6830	98.9	0.5548	0.6672	83.1	1.1387
**18.**	1.2098	1.5246	98.1	0.6494	0.7514	85.8	1.3538

^1^—plasma–protein binding log K predicted by Vega QSAR [35].

**Table 3:** Comparison of retention of investigated compounds statistics in various chromatographic systems.

Parameter	log k_w_ C-18	log k_w_ IAM	log k_w_ Chol	log k HSA	log k AGP	-S C-18	-S IAM	-S Chol
Median	2.698	1.705	3.003	1.363	0.507	4.742	6.093	5.032
Mean	2.700	1.799	2.971	1.350	0.509	4.750	6.069	4.873
Std. deviation	0.467	0.471	0.594	0.374	0.199	0.430	0.892	0.642
Range	1.918	1.583	2.304	1.094	0.634	1.877	3.165	2.431

**Table 4:** Log P (log D) parameters predicted in silico using different calculation algorithms.

No.	log P ^1^	C log P ^1^	log P (M-K) ^2^	M LogP ^3^	A Log P ^3^	S + logP ^4^	S + logD ^4^
**1.**	2.64	2.0362	1.77	2.34	2.73	2.173	2.171
**2.**	2.8	2.1792	1.97	2.73	2.93	2.511	2.508
**3.**	3.2	2.7492	2.42	2.86	3.39	2.747	2.742
**4.**	4.0	3.1592	2.94	3.10	3.31	3.111	3.108
**5.**	2.8	2.1792	1.97	2.73	2.93	2.506	2.503
**6.**	3.2	2.7492	2.42	2.86	3.39	2.774	2.77
**7.**	3.47	2.8992	2.66	2.98	3.48	2.867	2.864
**8.**	4.0	3.1592	2.94	3.10	3.31	3.138	3.136
**9.**	2.8	2.1792	1.97	2.73	2.93	2.446	2.443
**10.**	3.2	2.7492	2.42	2.86	3.39	2.759	2.756
**11.**	3.47	2.8992	2.66	2.98	3.48	2.86	2.858
**12.**	4.0	3.15916	2.94	3.10	3.31	3.157	3.156
**13.**	3.76	3.4622	3.06	3.37	4.06	3.388	3.380
**14.**	3.76	3.4622	3.06	3.37	4.06	3.432	3.423
**15.**	3.76	3.3422	3.06	3.37	4.06	3.407	3.401
**16.**	1.63	1.7792	1.59	2.35	2.62	2.268	2.263
**17.**	3.08	2.5952	2.32	2.86	3.27	2.648	2.646
**18.**	3.64	3.2102	2.95	3.15	3.64	3.193	3.19

^1^—log P models calculated by ChemDraw Ultra 10.0. software [38]. ^2^—log P model prediction by Vega QSAR [35]. ^3^—log P models prediction by Percepta 2.0 software [39]. ^4^—log P calculated by MedChem Designer 3.0.0.30 tools [40].

**Table 5:** Correlation matrix (r) of log k_w_ parameters obtained by HPLC using various stationary phases and log P parameters calculated using various computational approaches.

Descriptor	log k_w_ C-18	log k_w_ IAM	log k_w_ Chol	log K HSA	log K AGP
log P ^1^	0.84 (4, 16) ^5^	0.90 (4, 16) ^5^	0.84 (4, 16) ^5^	0.79 (4, 16) ^5^	0.83
C log P ^1^	0.87 (16) ^5^	0.87 (16) ^5^	0.84 (4, 16) ^5^	0.72 (7, 16) ^5^	0.92 (13) ^5^
S + log P ^2^	0.89 (16) ^5^	0.89 (18) ^5^	0.82 (4, 16) ^5^	0.68 (7) ^5^	0.93 (13) ^5^
S + log D ^2^	0.89 (16) ^5^	0.85	0.82 (4, 16) ^5^	0.68 (7) ^5^	0.92 (13) ^5^
M log P ^3^	0.90 (16) ^5^	0.85 (16) ^5^	0.81 (4, 16) ^5^	0.60 (7) ^5^	0.91 (13) ^5^
ALogP ^3^	0.89 (16) ^5^	0.81	0.82 (8, 16) ^5^	0.56	0.83 (13) ^5^
log P (M-K) ^4^	0.89 (4, 16) ^5^	0.83	0.86 (4, 16) ^5^	0.70 (16) ^5^	0.94 (13) ^5^

^1^—log P models calculated by ChemDraw Ultra 10.0. software [38]. ^2^—log P calculated by MedChem Designer 3.0.0.30 software [40]. ^3^—log P calculated by Percepta 2.0 [39]. ^4^—log P model prediction by Vega QSAR [35]. ^5^—outlier compounds.

## Data Availability

All data is available in ‘Supplementary Materials’ of this contribution.

## Supplementary Materials

The following supporting information can be downloaded at: https://www.mdpi.com/article/10.3390/molecules28207107/s1, log k values for C-18, IAM and Chol stationary phases for all mobile phases studied. Table S1: Log k values for all mobile phase compositions studied (MeOH/H_2_O, *v*/*v*) on C18 stationary phase; Table S2: Log k values for all mobile phase compositions (ACN/H_2_O, *v*/*v*) studied on IAM stationary phase; Table S3: Log k values for all mobile phase compositions (studied MeOH/H_2_O, *v*/*v*) on Chol stationary phase.

## Sample Availability

Samples of compounds **1**–**18** are available from the authors.
