# Supplementary material for: Retention Behavior of Anticancer Thiosemicarbazides in Biomimetic Chromatographic Systems and In Silico Calculations
Source: Molecules. 2023 Oct 16;28(20):7107. doi: 10.3390/molecules28207107 (PMC10608985; doi:10.3390/molecules28207107)
Supplement: Supplementary file 1 [file molecules-28-07107-s001.zip › molecules-2634227-supplementary.pdf]

Retention Behavior of Anticancer Thiosemicarbazides in Biomimetic Chromatographic Systems and In Silico Calculations

Marek Studziński [1], Paweł Kozyra [2], Monika Pitucha [2], Bogusław Senczyna [3] and Joanna Matysiak [3,*]

1 Department of Physical Chemistry, Institute of Chemical Sciences, Faculty of Chemistry, Maria Curie-Skłodowska University, 20-031 Lublin, Poland; marek.studzinski@umcs.pl
2 Independent Radiopharmacy Unit, Medical University of Lublin, Chodzki 4a, PL-20093 Lublin, Poland; pawelkozyra@umlub.pl (P.K.); monikapitucha@umlub.pl (M.P.)
3 Department of Chemistry, University of Life Sciences in Lublin, Akademicka 15, 20-950 Lublin, Poland; boguslaw.senczyna@up.lublin.pl
* Correspondence: joanna.matysiak@up.lublin.pl; Tel.: +48-814456816

Table S1. Log k values for all mobile phase compositions studied (MeOH/H_2O, v/v) on C 18 stationary phase

v/v, MeOH<br>compound	0.90	0.80	0.70	0.60	0.50	0.40
**1.**	-1.542	-1.263	-0.841	-0.508	-0.153	0.359
**2.**	-1.919	-1.346	-0.927	-0.530	-0.161	0.342
**3.**	-1.743	-1.202	-0.711	-0.327	0.095	0.574
**4.**	-1.477	-1.100	-0.579	-0.147	0.244	0.723
**5.**	-1.919	-1.343	-0.820	-0.406	0.017	0.537
**6.**	-1.624	-1.094	-0.579	-0.178	0.288	0.864
**7.**	-1.526	-0.986	-0.541	-0.109	0.367	0.967
**8.**	-1.446	-0.948	-0.463	-0.022	0.464	1.094
**9.**	-1.665	-1.491	-0.817	-0.455	-0.063	0.415
**10.**	-1.433	-1.029	-0.573	-0.171	0.311	0.842
**11.**	-1.367	-0.986	-0.507	-0.091	0.386	0.959
**12.**	-1.323	-0.934	-0.416	0.009	0.500	1.116
**13.**	-1.496	-0.901	-0.344	0.107	0.558	1.164
**14.**	-1.421	-0.769	-0.190	0.319	0.854	-
**15.**	-1.446	-0.881	-0.316	0.171	0.694	-
**16.**	-	-1.336	-0.976	-0.443	0.074	0.732
**17.**	-1.538	-1.063	-0.633	-0.142	0.364	0.788
**18.**	-1.547	-1.058	-0.626	-0.126	0.292	0.827

Table S2. Log k values for all mobile phase compositions (ACN/H_2O, v/v) studied on IAM stationary phase

v/v ACN compound	0.40	0.35	0.30	0.25	0.20	0.15	0.10	0.05
**1.**	-0.556	-0.490	-0.518	-0.142	0.085	0.284	0.548	0.999
**2.**	-	-0.461	-0.358	-0.010	0.234	0.455	0.692	0.968
**3.**	-	-0.209	-0.138	0.291	0.515	0.794	1.022	-
**4.**	-	-0.138	-0.029	0.451	0.642	0.893	1.158	-
**5.**	-	-0.441	-0.274	0.024	0.312	0.565	0.876	1.193
**6.**	-	-0.247	-0.082	0.333	0.647	0.982	1.270	-
**7.**	-	-0.196	-0.038	0.413	0.777	1.120	-	-
**8.**	-0.404	-0.074	0.088	0.573	0.952	1.304	-	-
**9.**	-	-0.467	-0.484	-0.150	0.088	0.336	0.588	1.054
**10.**	-	-0.210	-0.038	0.368	0.673	1.009	1.235	-
**11.**	-	-0.111	0.004	0.453	0.792	1.141	-	-
**12.**	-0.358	-0.016	0.141	0.655	0.951	1.352	-	-
**13.**	-0.430	-0.103	0.067	0.557	0.870	1.240	-	-
**14.**	-0.366	-0.027	0.154	0.710	1.133	1.494	-	-
**15.**	-0.346	-0.062	0.158	0.667	1.032	1.542	-	-
**16.**	-	-0.533	-	0.016	0.253	0.590	0.964	1.287
**17.**	-	-0.280	-0.101	0.282	0.513	0.821	1.147	-
**18.**	-	-0.258	-0.130	0.278	0.533	0.809	1.105	-

Table S3. Log k values for all mobile phase compositions (studied MeOH/H_2O, v/v) on Chol stationary phase

v/v, MeOH compound	0.80	0.70	0.60	0.50	0.45	0.40	0.35	0.30
**1.**	-1.006	-	-0.157	0.110	0.440	0.620	0.857	0.979
**2.**	-1.104	-	-0.314	-0.037		0.441	0.656	0.744
**3.**	-1.020	-	-0.068	0.198	0.461	0.759	0.917	1.024
**4.**	-0.938	-	-0.037	0.244	0.519	0.747	0.974	1.105
**5.**	-1.065	-	-0.057	0.184	0.487	0.699	0.957	-
**6.**	-0.975	-	0.160	0.422	0.766	1.016	1.270	-
**7.**	-0.987	-0.309	0.347	0.720	0.913	1.284	-	-
**8.**	-0.984	-0.165	0.522	0.834	1.026	1.443	-	-
**9.**	-0.997	-	-0.078	0.179	0.523	0.696	0.970	1.111
**10.**	-0.946	-0.396	0.206	0.479	0.836	1.050	1.331	1.475
**11.**	-0.886	-	0.276	0.571	0.871	1.158	1.437	-
**12.**	-0.783	-0.214	0.404	0.685	1.049	1.303	-	-
**13.**	-1.016	-0.329	0.400	0.627	0.978	1.246	-	-
**14.**	-0.783	-0.138	0.574	0.716	1.095	-	-	-
**15.**	-0.876	-0.087	0.792	1.013	1.349	-	-	-
**16.**	-	-	-0.189	0.093	0.478	0.843	1.082	1.264
**17.**	-0.975	-0.316	0.379	0.510	0.878	1.108	1.434	-
**18.**	-1.016	-	0.062	0.405	0.758	1.025	1.296	1.494
